# Risk of Sleepiness-Related Accidents in Switzerland: Results of an Online Sleep Apnea Risk Questionnaire and Awareness Campaigns

**DOI:** 10.3389/fmed.2017.00034

**Published:** 2017-04-12

**Authors:** Mona Lichtblau, Daniel Bratton, Philippe Giroud, Thomas Weiler, Konrad E. Bloch, Thomas Brack

**Affiliations:** ^1^Sleep Disorders Center, Department of Respiratory Medicine, University Hospital Zurich, Zurich, Switzerland; ^2^Swiss Lung League, Bern, Switzerland; ^3^Department of Internal Medicine, Cantonal Hospital Glarus, Glarus, Switzerland

**Keywords:** sleep apnea, survey, accidents, sleepiness, prediction model, web questionnaire

## Abstract

**Objectives:**

Obstructive sleep apnea syndrome (OSAS) is associated with major morbidity and mortality but OSAS is frequently under recognized. To promote awareness of OSAS, the Swiss Lung League launched an online questionnaire combined with annual advertisements in mass media. Characteristics of participants, prevalence of sleep apnea, OSAS-related symptoms, and their association with accidents were investigated.

**Methods:**

A questionnaire was created incorporating anthropometrics, the Epworth sleepiness scale (ESS), the OSAS domain of the sleep disorders questionnaire (SDQ), and a question on accidents related to sleepiness.

**Results:**

A total of 198,422 persons participated, 63% were male, mean (±SD) age was 45 (±16) years, weight 80 (±18) kg, height 173 (±9) cm, and body mass index 26.7 (±5.4) kg/m^2^. Some male (19%) and female (17%) participants had both elevated ESS and SAS scores (SAS > 35 (m)/SAS > 31 (f) and ESS > 10) and were suspected of having sleep apnea. In all, 6,654 (3.4%) had suffered an accident related to sleepiness. In multivariate regression analysis, ESS item #8 (falling asleep on the wheel, while stopping for a few minutes in traffic) was closest related to suffering an accident (OR 2.8).

**Conclusion:**

The OSAS awareness campaign of the Swiss Lung League reached a large number of people of both genders and of a wide age range. Many participants suffered from excessive sleepiness and symptoms of sleep apnea were highly prevalent. The campaign raised awareness of OSAS and contributed to the diagnosis and treatment of sleep apnea, thereby possibly preventing related morbidity and mortality.

## Introduction

Sleep apnea (SA) is a common disease in industrialized countries; recent findings from the HypnoLaus study report a prevalence of 23.4% in women and 49.7% in men in the Swiss general population (1), reflecting the results published by the Wisconsin Sleep Cohort Study that suggested a growth in prevalence of sleep-disordered breathing between 14 and 55% in the last decade (2). SA frequently causes excessive daytime sleepiness and may be the source of car accidents, threatening not only the life of the driver but also of other traffic participants (3–5). Furthermore, SA is associated with arterial hypertension and the metabolic syndrome, leading to increased cardiovascular morbidity such as left ventricular dysfunction, coronary artery disease, and stroke (6–8). Many patients with obstructive sleep apnea syndrome (OSAS) remain untreated because the general population lacks awareness of the disorder and its unfavorable consequences. Thus, better general knowledge of the disease and its associated risks is important.

In 2006, Schwegler et al. (9) published data from a sleep and wakefulness disturbance study of Swiss pharmacy customers. A cohort of 4,901 patients was asked to complete an internet-based questionnaire containing the Stanford Sleep Disorders Questionnaire (SDQ) and the Epworth Sleepiness Scale (ESS). Results showed a very high prevalence of daytime sleepiness and elevated scores in the sleep apnea questionnaire in this specific population, resulting in the idea of raising awareness for sleep disorders in Switzerland and to collect data from a more diverse study population. Therefore, an online risk assessment test for sleep apnea was created and made available on the website of the Swiss Lung League, a non-profit organization for patients with respiratory diseases. Annual advertisings including nationwide poster campaigns (2009–2013) and a TV advertisement (2014) were launched to increase attention for sleep apnea.

The purpose of this study was to evaluate the effectiveness of the media campaign and the online questionnaire on participation rate and to analyze the prevalence of sleepiness and other symptoms related to sleep apnea. We hypothesized that symptoms of sleep apnea and daytime sleepiness are highly prevalent in the Swiss general population. Furthermore, we evaluated single items of the questionnaires and their association with sleepiness-related accidents. Based on the results of multivariate regression analyses, we modeled the risk of sleepiness-related accidents.

## Materials and Methods

### Study Population

Between January 2009 and November 2014 visitors of the Swiss Lung League website were asked to answer the online questionnaire. Nationwide advertisements targeted snorers and people with daytime sleepiness. No consent for this study was required and feasible since the participants responded anonymously.

### Web-Based Questionnaire

The questionnaire was available on the website of the Swiss Lung League in three official Swiss languages (German, French, and Italian) and consisted of three pages with the first page asking for demographics (gender, age, height, and weight), smoking habits, arterial hypertension, and a question about having suffered sleepiness-related accidents. The user could only advance to the next page if all questions were answered. On the second page the participant answered the Epworth Sleepiness Scale (ESS) (10, 11), and the third page contained a subset of questions of the Sleep Apnea Subscale (SAS) from the Stanford SDQ (12), asking about sleep apnea symptoms such as snoring, breathing pauses during sleep, or difficulties breathing through the nose. After completing the questionnaire, the participant was provided with a summary of answers including the ESS and SAS Score and a computer-generated interpretation of the results. If the participant scored ESS > 10 and SAS > 35(m)/31(f), a message was generated telling that some answers indicated the possibility of sleep apnea syndrome or another sleep disorder, and a consultation with a physician was recommended. Results of the survey were stored in a database (TYPO3, Version 6.2.15). Multiple entries by the same individual could not be detected, since identification of participants was prevented to protect privacy. The questionnaire is available on the web page of the Swiss Lunge League: http://www.lungenliga.ch/de/krankheiten-ihre-folgen/schlafapnoe/diagnose/schlafapnoe-risikotest.html, the English version is presented in Table S1 in Supplementary Material.

### The Epworth Sleepiness Scale (ESS)

The ESS investigates the tendency to fall asleep in eight different everyday situations. Each item is rated on a scale ranging from 0 (unlikely to fall asleep) to 3 (very likely to fall asleep). The sum of all items (0–24) is a measure of daytime sleepiness (10, 11). A score >10 indicates excessive daytime sleepiness.

### The Sleep Apnea Subscale (SAS) of the SDQ

Douglass et al. (12) found a subset of 12 questions from the SDQ to be the most reliable to test for sleep apnea. These 12 items were used in the current questionnaire (12, 13), and each item was rated on a scale ranging from 1 (never) to 5 (always). Additionally to sleep apnea symptoms, age, weight, body mass index (BMI), smoking habits, and blood pressure are also taken into account. A score >35 for males and >31 for females indicates a high probability of suffering from sleep apnea or other sleeping disorders (12).

### Data Analysis and Statistics

The dataset was imported to Statistica (Version 8, StatSoft, Tulsa, USA) and checked for completeness; labels and parsing were checked. Plausibility tests were also performed; only complete questionnaires from participants with an age of 16–99 years, a weight of 35–180 kg and a height of 140–205 cm were accepted for calculation. Patient characteristics are presented as mean and SD. Due to the large sample size, normality was assumed and *t*-tests were used to compare means between groups.

Sleep Apnea was suspected if cutoff values of both questionnaires were surpassed [SAS > 35 (male)/SAS > 31 (female) and ESS > 10]. Patients with only one elevated score were not classified as SA for the analysis, but they were still advised to see a physician for further investigation. Cronbach’s Alpha was calculated for the SAS of the SDQ and ESS to test for internal consistency of the scales.

A multivariable logistic regression model was used to determine the association between accidents caused by sleepiness (binary outcome) and the following independent variables: gender, smoking history, high blood pressure, each item of the ESS and SAS (categorical covariates), BMI, and age (continuous covariates).

A prediction model and a risk calculator for accidents were generated. To compute the prediction model, we first fitted the logistic regression model with accidents caused by sleepiness as the outcome and age, sex, BMI, smoking status, high blood pressure, and each component of the ESS and SAS as covariates (“Full model”). The associations of age and BMI with accidents were first determined using separate multivariable second degree fractional polynomial models (FP2) adjusting for the categorical variables (14). The best fitting FP2 transformations of age and BMI were then included in the main model. The preferred model was that which minimized the Bayesian information criterion (BIC) (15). Starting with the full model, each covariate was removed from the model and that which led to the greatest reduction in the BIC when removed was rejected. This process was then repeated until removing covariates from the model did not lead to any further reductions in the BIC.

The discriminative ability of the final model was assessed using 10-fold cross-validation. Data were split by the deciles of the date in which the questionnaire was completed. Discrimination in each group was assessed using the area under the receiver operating characteristic (ROC) curve using the model built on data from the other nine groups. For comparison, we also assessed the discrimination of the model at each step of the selection procedure described above, including the full model and the final model without the ESS components. The calibration of the prediction model, which had the highest area under the ROC curve in the cross-validation, was assessed by comparing the predicted risk of accidents to the actual proportion of accidents in groups defined by predicted risk. Regression analysis and prediction model was carried out with Stata Version 11.

## Results

### Study Population and Demographics

Between January 2009 and November 2014, 199,161 participants completed the online survey of which 739 (0.4%) did not meet plausibility criteria leaving 198,422 surveys for analysis. In all, 62.9% of the participants were male; mean (±SD) age was 45 (±16) years, weight 80 (±18) kg, height 173 (±9) cm, and BMI 26.7 (±5.4) kg/m^2^ (Table 1). Out of 49% of participants who smoked, 25% were heavy smokers with a history of more than 25 years of smoking. Arterial hypertension was indicated by 23% of the participants, while 14% did not know if they had high blood pressure.

**Table 1:** **Characteristics of overall participants, of respondents with and without elevated scores suggesting sleep apnea, and of participants who caused accidents compared to those who did not cause accidents**.

	Sex	No. of subjects	Age, years	Weight, kg	Height, cm	BMI, kg/m^2^	SAS	ESS
Overall participants	All	198,422	45 ± 16	80.3 ± 18.4	173 ± 9	26.7 ± 5.4	32.2 ± 8.4	8.9 ± 4.5
	M	124,758	47 ± 15	86.2 ± 16.2	178 ± 7	27.2 ± 4.8	34.3 ± 7.9	8.7 ± 4.4
	F	73,664	42 ± 16	70.3 ± 17.5	165 ± 6	25.7 ± 6.3	28.8 ± 8.1	9.2 ± 4.5
Suspects of sleep apnea^a^	M	23,502	49 ± 13	93.3 ± 17.4	178 ± 7	29.5 ± 5.2	42.4 ± 4.8	14.2 ± 2.9
	F	12,222	46 ± 14	79.4 ± 19.3	165 ± 7	29.1 ± 7.0	38.2 ± 5.0	14.3 ± 2.9
Healthy participants^b^	M	101,256	46 ± 16	84.6 ± 15.4	178 ± 7.0	26.7 ± 4.5	32.4 ± 7.2	7.4 ± 3.7
	F	61,442	42 ± 16	68.5 ± 16.5	165 ± 6	25.1 ± 5.9	26.9 ± 7.3	8.2 ± 4.1
Sleep apnea vs healthy^c^	M		2.7 (2.5; 2.9)*	8.7 (8.5; 8.9)*	−0.2 (−0.3; −0.1)*	2.8 (2.8; 2.9)*		
	F		4.2 (3.9; 4.5)*	10.8 (10.5; 11.2)*	−0.2 (−0.3; −0.1)^#^	4.0 (3.9; 4.2)*		
No accidents	All	191,768	45 ± 16	80.3 ± 18.3	173 ± 9	26.7 ± 5.4	32.1 ± 8.4	8.8 ± 4.4
Accidents	All	6,654	44 ± 16	81.9 ± 19.7	174 ± 9	27.0 ± 6.0	34.7 ± 9.2	12.2 ± 5.2
Accidents vs no accidents^d^	All		−1.3 (−1.7; −0.9)*	1.6 (1.3; 2.1)*	0.8 (0.5; 1.0)*	0.3 (0.2; 0.5)*	2.6 (2.4; 2.8)*	3.4 (3.3; 3.5)*

*^a^Suspects of sleep apnea = male: SAS > 35, ESS > 10, female: SAS > 31, ESS > 10*.

*^b^Healthy participants = male: SAS ≤ 35 or ESS ≤ 10, female: SAS ≤ 31 or ESS ≤ 10*.

*^c^Comparison of sleep apnea suspects and healthy controls (difference of means)*.

*^d^Comparison of groups (no accidents vs accidents) (difference of means)*.

*Values are given as mean ± SD or difference of means (95% CI). M, male; F, female participants; BMI, body mass index; SAS, Sleep Apnea Subscale of the Sleep-Disordered Questionnaire, ESS, Epworth Sleepiness Scale*.

*p < 0.000, ^#^p < 0.01.

**Figure 1:**
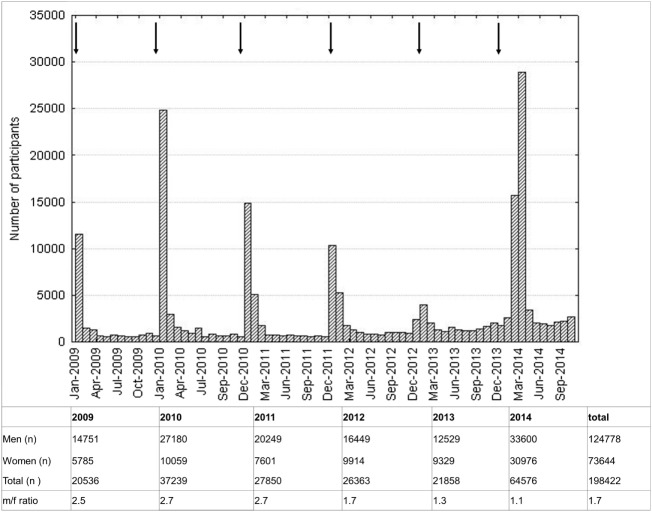
**Histogram of participation rate**. One bar represents 1 month. Arrows indicate advertisements.

The male to female ratio of participants was 1.7. At least twice as many participants were recruited in 2014 compared to other years, following a nationwide TV-ad in January 2014. Higher participation rates were observed during and two months after each nationwide campaign with advertisements on public places and in newspapers (Figure 1).

### SAS and ESS—Suspected Sleep Apnea

Mean (±SD) ESS was 8.9 (±4.5) for all participants, men scored 8.7 (±4.4) and women 9.2 (±4.5). Overall mean SAS was 32.2 (±8.4), for men 34.3 (±7.9) and for women 28.8 (±8.1), reflecting the known gender difference (Table 1) (12). A total of 68,436 (34.5%) participants had an elevated ESS and 82,513 (41.6%) an elevated SAS. A total of 35,724 (18%) of all participants (19% of the male (*n* = 23,502) and 17% of the female (*n* = 12,222) participants) reached the threshold in both the ESS and SAS questionnaires and were classified as suspected SA. Compared to the healthy participants, SA suspects were older and heavier, as shown in Table 1.

Cronbachs alpha was calculated to test for internal consistency of the questionnaires. For ESS items Cronbach α was 0.80 and for SAS items 0.74 (Table S2 in Supplementary Material). No increase in Cronbach’s alpha was revealed if any item was deleted.

### Accidents

A total of 6,654 participants (3.4%) reported suffering an accident due to sleepiness; 4,733 were male (3.8% of the male participants) and 1,921 female (2.6% of the female participants). Higher scores in SAS and ESS were found in subjects who suffered accidents (mean difference (95% CI) SAS: 2.6 (2.4–2.8) and ESS: 3.4 (3.3–3.5), all *p* < 0.001) (Table 1). Significant differences could also be found for the items of both questionnaires (all items *p* < 0.0001), except for smoking (SAS #10, *p* = 0.31).

A multivariate model was generated (Table S3 in Supplementary Material), and odds ratios for accidents were calculated for each item of the ESS and SAS questionnaires. Almost all items for the ESS except for item #2 (watching TV) and #5 (lying down to rest in the afternoon when circumstances permit) were associated with accidents while in the SAS only items #1 (sweat at night), #2 (nose blocks up while trying to sleep), #5 (snoring/breathing worse with alcohol), and #7 (waking up and being unable to breathe) were related to an increased risk of accidents. Male sex was associated with higher risk for suffering sleepiness-related accidents.

**Figure 2:**
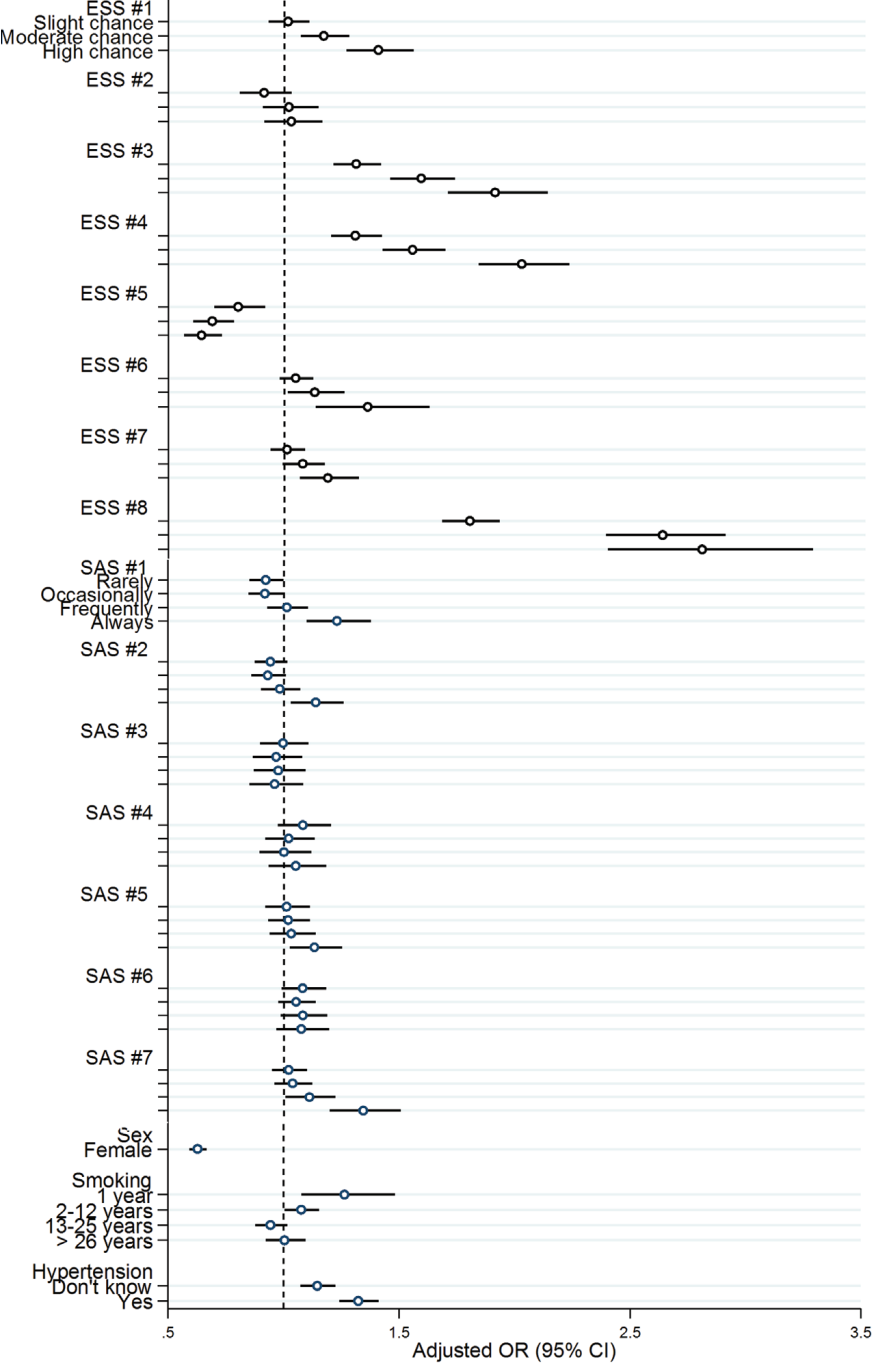
**Multivariable logistic regression: odds ratio and 95% confidential interval for items of the Epworth Sleepiness Score (ESS), the Sleep Apnea Subscale (SAS), gender, smoking history (SAS #10), and arterial hypertension (SAS #9)**. Dependent variable: accidents. ESS #1, sitting and reading; ESS #2, watching TV; ESS #3, sitting inactive in a public place (e.g., a theater or a meeting); ESS #4, as a passenger in a car for an hour without a break; ESS #5, lying down to rest in the afternoon when circumstances permit; ESS #6, sitting and talking to someone; ESS #7, sitting quietly after a lunch without alcohol; ESS #8, in a car, while stopped for a few minutes in traffic. SAS #1, sweat at night; SAS #2, nose blocks up while trying to sleep; SAS #3, snore that bothers others; SAS #4, snoring/breathing worse if sleeping on back; SAS #5, snoring/breathing worse with alcohol; SAS #6, stop breathing during sleep; SAS #7, suddenly wake up at night struggling for air, unable to breath.

The highest odds ratios were found for ESS #3 (sitting inactive in a public place), ESS #4 (traveling as a passenger in a car for an hour without a break), and ESS #8 (falling asleep in a car, while stopping for a few minutes in traffic) (OR in comparison to 0 points (would never doze): ESS #3: 1 point (slight chance of dozing): OR 1.3, 2 points (moderate chance of dozing): OR 1.6, 3 points (high chance of dozing): OR 1.9. ESS #4: 1 point: OR 1.3, 2 points: OR 1.6, 3 points: OR 2.0. ESS #8: 1 point: OR 1.8, 2 points: OR 2.6, 3 points: OR 2.8.) (Figure 2; Table S3 in Supplementary Material).

### Prediction Model for Accidents and Risk Calculator

**Figure 3:**
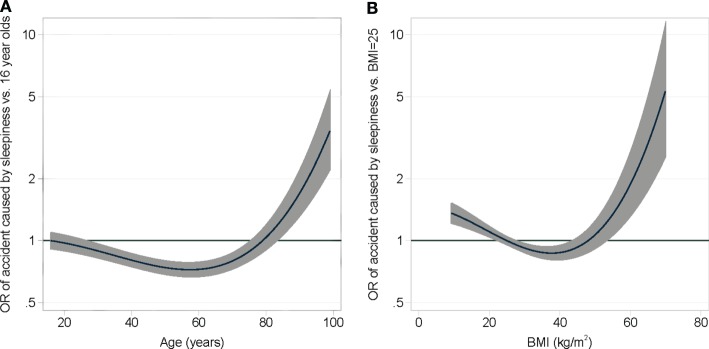
**A multivariable fractional second degree polynomial model adjusting the risk of causing an accident due to daytime sleepiness depending on age (A) and BMI (B)**.

A prediction model was generated from the results of the multivariate regression analysis. The model found no correlation of the OSAS symptoms of the SAS and the smoking history; therefore, these parameters were not integrated in the model. The covariates of the final model were sex, high blood pressure, all ESS items, and the second degree fractional polynomial transformations of age and BMI (Figure 3). A cross-validation was performed to assess the discriminative ability of the model. The Baesian information criterion and the results of the cross-validation assessment of each model are shown in Table S4 in Supplementary Material. The median area under the ROC curve of the final model was 0.73. Removing the components of the SAS and smoking history had a negligible impact on the discriminative ability of the final model compared to the full model. In contrast, removing all components of the ESS greatly reduced the area under the ROC curve, showing that the ESS is a strong predictor of accidents related to sleepiness. The risk of accidents can be calculated by the following formula:

risk (%)=(exp(s)/1+exp(s))×100

while *S* is the sum score of coefficients given in Table S5 in Supplementary Material and the excel calculator file in the Supplementary Material (*S* = −3.803897 + 0.4922852 × male − 0.0009926 × BMI^2^+…) (Table S5 in Supplementary Material, Risk Calculator in Data Sheet 1 in Supplementary Material).

For a male patient, 75 years, 120 kg, 180 cm with arterial hypertension and an ESS of 24 the risk calculator would predict a 37.8% chance of suffering an accident. Further exemplary calculations can be found in Table 2.

**Table 2:** **Examples of answers and their risk of causing an accident according to the risk calculator**.

Gender	Age, years	Weight, kg	Height, cm	BMI, kg/m^2^	ESS total score	ESS								Art. hypertension	Risk, %
						#1	#2	#3	#4	#5	#6	#7	#8		
F	20	55	167	19.7	5	0	1	0	1	3	0	0	0	No	1.2
M	68	80	179	25	6	0	3	1	0	2	0	0	0	No	1.7
M	38	94	188	26.6	13	2	2	1	3	3	0	0	2	No	9.7
M	62	93	182	28.1	20	3	3	3	2	3	1	3	2	No	14.5
M	57	155	184	45.8	23	3	3	3	3	3	2	3	3	Yes	28.1
M	75	120	180	37.0	24	3	3	3	3	3	3	3	3	Yes	37.8

*ESS, Epworth Sleepiness Scale: chance of falling asleep while: ESS#1, sitting and reading; ESS#2, watching TV; ESS#3, sitting inactive in a public place (e.g., theater, meeting, lecture); ESS#4, as a passenger in a car for an hour without a break; ESS #5, lying down to rest in the afternoon when circumstances permit; ESS #6, sitting and talking to someone; ESS#7, sitting quietly after a lunch without alcohol; ESS #8, in a car, while stopped for a few minutes in the traffic*.

## Discussion

In this study, we described the prevalence of sleep-apnea-related symptoms and daytime sleepiness in a large group of Swiss participants by means of the SAS of SDQ and ESS. The elevated scores in many of the participants suggest that sleep apnea is highly prevalent in the studied population and associated with accidents related to sleepiness. From our database, a prediction model was computed and a calculator was generated to forecast the risk of suffering a sleepiness-related accident.

Overall, 198,422 questionnaires were evaluated with an increasing participation rate in the weeks following nationwide poster and TV advertisements. Of all participants, 19% of the male and 17% of the female participants were suspected of suffering from SA according to cutoff values by Douglass and Johns (11, 12) and therefore were advised to seek professional medical consulting.

Even though the prevalence of SA in participants of this survey is probably overestimated due to attraction of subjects with SA-related symptoms, our data are nevertheless consistent with an increasing prevalence of SA in the general population during the past 15 years. In 2000, a prevalence of SA of about 6% in the employed Swiss population was reported (16). In 2015, the HypnoLaus Study reported a prevalence of sleep-disordered breathing in Switzerland of 23.4% in women and 49.7% in men diagnosed by polysomnography (PSG) with a threshold of an apnea-hypopnea index (AHI) ≥ 15/min in 2,121 participants from a population register of the city of Lausanne, Switzerland (age 40–85 years, mean BMI 26.2 kg/m^2^) (1). Furthermore the Sao Paulo Epidemiologic Sleep Study reported a prevalence of 30.5% in women and 46.6% in men in 1,042 participants (all underwent PSG age range 20–80 years) (17) and Duran and colleagues from Spain reported a prevalence of 28.0% in women and 26.2% in men in 2,148 participants (555 underwent PSG, age range 30–70 years) (18); both studies used an AHI > 5 as cutoff value for diagnosis of SA. The impressive increase of the prevalence of SA in the past 10 years may be due to the surge of obesity.

The internal consistency of SAS of the SDQ (Cronbach’s α = 0.74) and of the ESS (Cronbach’s α = 0.8) was comparable to earlier studies (9, 10, 12, 13). Although the ESS has shown a poor correlation with OSAS (1, 19), it was used in this questionnaire due to the fact that daytime sleepiness is one of the severe consequences of OSAS and highly correlated with accidents (4).

To elucidate the sequelae of suspected OSA, participants of the survey were asked if they had suffered accidents related to sleepiness. Of 198,422 participants, 6,654 (3.4%) reported a sleepiness-related accident. Scores of the ESS (8.8 vs 12.2) and SAS (31.2 vs 34.7) were higher in those causing accidents. Multivariable regression indicated male gender (OR 1.56), high blood pressure (OR 1.33), and scores in the items of the ESS #3, 4, 8 [falling asleep while: ESS #3; sitting inactive in a public area (OR 1.9); ESS #4 as a passenger in a car for an hour without break (OR 2.04), ESS #8 in a car, while stopping for a few minutes in traffic (OR 2.83)] to be independent predictors for suffering accidents. Our prediction model revealed that ESS in combination with age, gender, BMI, and blood pressure are strong predictors for sleepiness-related accidents with a median AUC of the ROC curve of 0.73. Including the SAS failed to improve the accuracy of the model, thus severity of OSAS might not influence sleepiness-related accidents. Other risk factors related to OSA associated road accidents, e.g., collar size, driving habits, and yearly mileages, were not available for risk calculation but may nevertheless be important.

Maycock has previously described significant correlations between daytime sleepiness and accidents in UK car drivers (20), assessing 4,621 questionnaires that were answered by male drivers from the national Driver and Vehicle Licensing Agency’s driver file (response rate 51.3%) with a mean age of 48 years and an average annual mileage of 11,378 mi. It was found that daytime sleepiness was a much more reliable predictor when combined with other factors such as young age, occupational driving with high annual mileages, and snoring as compared to ESS alone: the risk of causing an accident for a company car driver with an ESS of 12 who feels close to falling asleep behind the wheel and does not regularly snore is 70% higher than for a driver with the same driving and snoring habits but with an ESS of 0 points (20). In another publication, 966 randomly sampled heavy good vehicle drivers (mean age 41 years, annual driving average 69,700 mi) at a motorway service area reported a total of 241 accidents over 3 years. In those drivers, the best prediction model for accidents contained ESS, snoring habits, and collar size, resulting in a 2.49 times higher accident liability in snorers with a large collar size and an ESS of 11 than in those with the same snoring habits and the same collar size but with an ESS of 0. Interestingly, ESS did not have an influence on accident liability in those who did not snore and had a small collar size (beta coefficient 0.006) (21).

A European wide web-based survey, investigating 12,434 questionnaires from 19 countries, showed similar determinants for sleepiness-related accidents as our study and the above mentioned studies have revealed: younger age (OR 1.56), male gender (OR 1.79), annual driving mileage ≥20,000 km (OR 2.02), daytime sleepiness (OR 7.49), and obstructive sleep apnea (3.48) (22). However, objective data on sleep apnea are missing since in none of the abovementioned investigations sleep studies were performed. Recently, a study conducted in Japan investigated 2,387 subjects that underwent PSG and completed a questionnaire on sleepiness-related driving accidents and the ESS, revealing a significantly higher risk for accidents caused by daytime sleepiness in patients with very severe OSAS (AHI > 60) and severe OSAS (30 < AHI < 60) compared to groups of simple snorers (16.9 vs 9.8 vs 6.4%). For subjects that reported accidents related to tiredness, the AUC of the ROC for the ESS was 0.672 and an ESS of 16 was found to be the best cutoff value for predicting accidents (specificity 0.895, sensitivity 0.263) (23).

Karimi et al. found patients with OSAS to have a 2.45 higher risk for accidents when compared to a control population, singling out the oldest population (65–80 years) of having the highest risk with an OR of 3.5. They also found an increased risk in patients with high annual mileage (>10,000 km/years, OR 1.1), high ESS (≥16, OR 2.1), short sleeping time (≤5 h/night, OR 2.7), and use of hypnotics (OR 2.1). CPAP therapy (≥4 h/night) reduced the risk from 7.6 to 2.5 accidents/1,000 drivers/years. However, the AHI did not correlate with accident risk, suggesting that other factors play an important role in tiredness-related accidents (24). The latter finding may explain why the SAS did not correlate with accidents in our multivariate regression analysis and prediction model.

Our study has some limitations. Excessive daytime sleepiness detected by the ESS can be caused by many different factors other than OSAS. By combining the ESS with the SAS we intended to improve the specificity for identification of OSAS. However, this could not be validated by sleep studies. Also, the yearly advertisement for the study clearly addressed snorers and people with daytime sleepiness, therefore a selection bias is likely and the data do not represent actual prevalence of sleep apnea symptoms in Switzerland. Multiple entries by the same individual could not be excluded, but we assume that this occurred in only a small fraction of all participants.

In our analysis, we only included patients that had both an elevated SAS and ESS so that we might have missed some patients with mild OSAS with only one of the two scores reaching the threshold for suspected OSA.

The phrasing of the question about accidents is another limiting factor of this study because the question did not distinguish between different types of accidents; neither did it account for repeated accidents. Furthermore, driving habits and hours were not assessed, which could be a major cofounder for risk of accidents as found by Maycock and Goncalves (20–22).

The OSAS awareness campaign of the Swiss Lung League reached a large number of persons of both genders and of a wide age range. A large number of participants suffered from excessive daytime sleepiness (Epworth score > 10) and symptoms of sleep apnea were highly prevalent. Sleep-disordered breathing and daytime sleepiness were associated with health risks including traffic accidents and cardiovascular diseases. Raising public awareness may contribute to the diagnosis and treatment of sleep apnea, thereby preventing its potentially hazardous consequences.

## Author Contributions

KB and ML: conception and design, data acquisition, analysis, interpretation, drafting the manuscript, revising it critically for important intellectual content, final approval, and agree to be accountable for all aspects of the work including accuracy and integrity are appropriately investigated and resolved. TW and PG: conception and design, data acquisition, analysis, interpretation, revising the manuscript critically for important intellectual content, final approval, and agree to be accountable for all aspects of the work including accuracy and integrity are appropriately investigated and resolved. DB and TB: analysis, interpretation, drafting the manuscript, revising it critically for important intellectual content, final approval, and agree to be accountable for all aspects of the work including accuracy and integrity are appropriately investigated and resolved.

## Conflict of Interest Statement

The authors declare that the research was conducted in the absence of any commercial or financial relationships that could be construed as a potential conflict of interest.
