# Supplementary material for: Risk of Sleepiness-Related Accidents in Switzerland: Results of an Online Sleep Apnea Risk Questionnaire and Awareness Campaigns
Source: Front Med (Lausanne). 2017 Apr 12;4:34. doi: 10.3389/fmed.2017.00034 (PMC5388690; doi:10.3389/fmed.2017.00034)
Supplement: Supplementary file 1 [file Table_1.DOCX]

**Table S1**

**The Web based Questionnaire (English Version)**

1. **Questions about personal information**

**Your gender**

5 = female 0 = male  5  0

**Your age** (this test is intended for people age 16 or older)

1 = 16–25 years 3 = 36–44 years 5 = 51 years or older

2 = 26–35 years 4 = 45–50 years  1  2  3  4  5

**Your current weight**

1 = 60 kg or less 3 = 73–84 kg 5 = 95 kg or more

2 = 61–72 kg 4 = 85–94 kg  1  2  3  4  5

**Do you currently have or had high blood pressure?**

1 = no 3 = don’t know 5 = yes  1  3 5

**Are you or have you been a smoker?**

**For how many years did you smoke or have you been smoking?**

| 1 = non-smoker | 3 = 2–12 years | 5 = 26 years or longer |  |
| --- | --- | --- | --- |
| 2 = 1 year | 4 = 13–25 years |  |  1  2  3  4  5   2   3   4   5 |

**Have you ever caused an accident due to sleepiness?**  yes  no

1. **Questions about daytime sleepiness (Epworth Sleepiness Scale)**

**How likely are you to doze off or fall asleep in the following situations?**

The following questions evaluate whether you actually doze off or fall asleep, in contrast to just feeling tired. This refers to your usual way of life in the past few weeks. Even if you haven’t done some of these things recently try to work out how they would have affected you.

Use the following scale to choose the most appropriate number for each situation:

Score:

0 = would never doze

1 = slight chance of dozing

2 = moderate chance of dozing

3 = high chance of dozing

**Situation**

**Chance of dozing**

| Sitting and reading |  0 |  1 |  2 |  3 |
| --- | --- | --- | --- | --- |
| Watching TV |  0 |  1 |  2 |  3 |
| Sitting inactive in a public place (e.g. theatre, meeting, lecture) |  0 |  1 |  2 |  3 |
| As a passenger in a car for an hour without a break |  0 |  1 |  2 |  3 |
| Lying down to rest in the afternoon when circumstances permit |  0 |  1 |  2 |  3 |
| Sitting and talking to someone |  0 |  1 |  2 |  3 |
| Sitting quietly after a lunch without alcohol |  0 |  1 |  2 |  3 |
| In a car, while stopped for a few minutes in the traffic |  0 |  1 |  2 |  3 |
| **Total score:** |  |  |  |  |
| **Interpretation** |  |  |  |  |

0 –10 points: No increased sleepiness.

11–14 points*: Slightly increased sleepiness.

15 points or more*: Considerably increased sleepiness.

* Increased sleepiness may have various causes (e.g. insufficient sleep, unfavourable sleeping habits, sleep disorders including sleep apnoea syndrome). If sleepiness interferes considerably with your daily activities, we recommend you to consult your doctor.

1. **Questions about snoring/sleep (SAS questionnaire)**

**The following questions refer to the past six months. Which of these statements describe your condition?**

Use the following scale to choose the most appropriate number for each question:

| 1 = never (not true at all)  2 = rarely (hardly true) | 3 = occasionally (don’t know)  4 = frequently (fairly true) | 5 = always (exactly true) | | | |  |
| --- | --- | --- | --- | --- | --- | --- |
| I am sweating much during the night. | |  1 |  2 |  3 |  4 |  5 |
| When going to sleep, I have difficulties breathing through the nose. | |  1 |  2 |  3 |  4 |  5 |
| I have been told that I snore loudly/my snoring disturbs others. | |  1 |  2 |  3 |  4 |  5 |
| When lying on my back, my snoring is clearly louder and/or my breathing problems are clearly worse. | |  1 |  2 |  3 |  4 |  5 |
| When I have had alcohol before going to sleep, my snoring is clearly louder and/or my breathing problems are clearly worse. | |  1 |  2 |  3 |  4 |  5 |
| I have been told that my breathing transiently stops during sleep. | |  1 |  2 |  3 |  4 |  5 |
| I suddenly wake up at night struggling for air, unable to breathe. | |  1 |  2 |  3 |  4 |  5 |
| **Total score:** | |  |  |  |  |  |

Note: In the online version of the Sleep Apnoea Risk Test (www.lungenliga.ch), body mass index (BMI)

is additionally included in the assessment.

**Interpretation**

0–35 points: Your answers do not suggest that you may suffer from sleep apnoea syndrome.

36 points or more: Some of your answers suggest that you may suffer from sleep apnoea syndrome. We recommend that you to consult your doctor
