# Supplementary material for: Risk of Sleepiness-Related Accidents in Switzerland: Results of an Online Sleep Apnea Risk Questionnaire and Awareness Campaigns
Source: Front Med (Lausanne). 2017 Apr 12;4:34. doi: 10.3389/fmed.2017.00034 (PMC5388690; doi:10.3389/fmed.2017.00034)
Supplement: Supplementary file 2 [file Table_2.DOCX]

| **Table S2** Item analysis of the Epworth Sleepiness Scale (ESS) and Sleep Apnea Score (SAS) of the Sleep Disorder Questionnaire (SDQ) | | |
| --- | --- | --- |
| Item number ESS | item to total correlation | Cronbach α if deleted |
| ESS1 Sitting and reading | 0.58 | 0.77 |
| ESS2 Watching TV | 0.44 | 0.79 |
| ESS3 Sitting inactive in a public place (e.g. a theater or a meeting) | 0.62 | 0.76 |
| ESS4 As a passenger in a car for an hour without a break | 0.55 | 0.77 |
| ESS5 Lying down to rest in the afternoon when circumstances permit | 0.41 | 0.79 |
| ESS6 Sitting and talking to someone | 0.51 | 0.78 |
| ESS7 Sitting quietly after a lunch without alcohol | 0.59 | 0.76 |
| ESS8 In a car, while stopped for a few minutes in traffic | 0.46 | 0.79 |
| n = 198422, Cronbach α = 0.80 |  |  |
|  |  |  |
| Item number SAS | item to total correlation | Cronbach α if deleted |
| SAS1 Sweat at night | 0.20 | 0.74 |
| SAS2 Nose blocks up while trying to sleep | 0.29 | 0.73 |
| SAS3 Snore that bothers others | 0.63 | 0.69 |
| SAS4 Snoring/breathing worse if on back | 0.60 | 0.70 |
| SAS5 Snoring/breathing worse with alcohol | 0.52 | 0.71 |
| SAS6 Stop breathing in sleep | 0.53 | 0.71 |
| SAS7 Awake unable to breath | 0.32 | 0.73 |
| SAS8 BMI | 0.44 | 0.72 |
| SAS9 High blood pressure | 0.24 | 0.75 |
| SAS10 Smoking | 0.17 | 0.75 |
| SAS11 Age | 0.21 | 0.75 |
| SAS12 Weight | 0.45 | 0.72 |
| n = 198422, Cronbach α = 0.74 |  |  |
