# Supplementary material for: Risk of Sleepiness-Related Accidents in Switzerland: Results of an Online Sleep Apnea Risk Questionnaire and Awareness Campaigns
Source: Front Med (Lausanne). 2017 Apr 12;4:34. doi: 10.3389/fmed.2017.00034 (PMC5388690; doi:10.3389/fmed.2017.00034)
Supplement: Supplementary file 3 [file Table_3.DOCX]

| Table S3 Multivariable binary logistic regression analysis. | | | | |
| --- | --- | --- | --- | --- |
| Dependent Variable: Accidents |  | | |  |
|  |  | | |  |
| Independent Variables | OR | 95%CI | | p-value |
|  |  |  |  |  |
| Age | 1.00 | 0.99 | 1.00 | <0.001 |
| BMI | 0.99 | 0.99 | 1.00 | 0.001 |
| Gender (0=m, 1=f) | 0.64 | 0.60 | 0.68 | <0.001 |
| ESS #1 Sitting and reading | | | | |
| 1 = slight chance of dozing (compared to 0 = would never doze) | 1.02 | 0.94 | 1.12 | 0.608 |
| 2 = moderate chance of dozing (compared to 0 = would never doze) | 1.18 | 1.08 | 1.29 | <0.001 |
| 3 = high chance of dozing (compared to 0 = would never doze) | 1.43 | 1.29 | 1.58 | <0.001 |
| ESS #2 Watching TV | | | | |
| 1 | 0.91 | 0.80 | 1.03 | 0.132 |
| 2 | 1.01 | 0.90 | 1.14 | 0.833 |
| 3 | 1.01 | 0.90 | 1.14 | 0.827 |
| ESS #3 Sitting inactive in a public place (e.g a theater or a meeting) | | | | |
| 1 | 1.31 | 1.21 | 1.41 | <0.001 |
| 2 | 1.58 | 1.45 | 1.73 | <0.001 |
| 3 | 1.90 | 1.70 | 2.13 | <0.001 |
| ESS #4 As a passenger in a car for an hour without a break | | | | |
| 1 | 1.31 | 1.20 | 1.42 | <0.001 |
| 2 | 1.56 | 1.43 | 1.70 | <0.001 |
| 3 | 2.04 | 1.85 | 2.24 | <0.001 |
| ESS #5 Lying down to rest in the afternoon when circumstances permit | | | | |
| 1 | 0.80 | 0.70 | 0.92 | 0.001 |
| 2 | 0.69 | 0.61 | 0.78 | <0.001 |
| 3 | 0.64 | 0.57 | 0.73 | <0.001 |
| ESS #6 Sitting and talking to someone | | | | |
| 1 | 1.05 | 0.98 | 1.12 | 0.208 |
| 2 | 1.13 | 1.01 | 1.25 | 0.031 |
| 3 | 1.39 | 1.17 | 1.67 | <0.001 |
| ESS #7 Sitting quietly after a lunch without alcohol | | | | |
| 1 | 1.02 | 0.94 | 1.10 | 0.635 |
| 2 | 1.09 | 1.00 | 1.18 | 0.048 |
| 3 | 1.20 | 1.08 | 1.34 | 0.001 |
| ESS #8 In a car, while stopped for a few minutes in traffic | | | | |
| 1 | 1.79 | 1.67 | 1.92 | <0.001 |
| 2 | 2.60 | 2.36 | 2.87 | <0.001 |
| 3 | 2.83 | 2.42 | 3.31 | <0.001 |
| SAS #1 Sweat at night | | | | |
| 2 = rarely (hardly true) (compared to 1 = never (not true at all)) | 0.92 | 0.85 | 0.99 | 0.035 |
| 3 = occasionally (don’t know) (compared to 1 = never (not true at all)) | 0.91 | 0.84 | 0.99 | 0.026 |
| 4 = frequently (fairly true) (compared to 1 = never (not true at all)) | 1.00 | 0.92 | 1.10 | 0.930 |
| 5 = always (exactly true) (compared to 1 = never (not true at all)) | 1.23 | 1.10 | 1.38 | <0.001 |
| SAS #2 Nose blocks up while trying to sleep | | | | |
| 2 | 0.94 | 0.88 | 1.02 | 0.131 |
| 3 | 0.94 | 0.86 | 1.02 | 0.115 |
| 4 | 0.99 | 0.91 | 1.08 | 0.871 |
| 5 | 1.16 | 1.05 | 1.28 | 0.004 |
| SAS #3 Snore that bothers others | | | | |
| 2 | 0.98 | 0.88 | 1.09 | 0.676 |
| 3 | 0.94 | 0.84 | 1.05 | 0.285 |
| 4 | 0.94 | 0.84 | 1.05 | 0.264 |
| 5 | 0.92 | 0.81 | 1.03 | 0.159 |
| SAS #4 Snoring/breathing worse if on back | | | | |
| 2 | 1.07 | 0.96 | 1.19 | 0.202 |
| 3 | 1.01 | 0.91 | 1.13 | 0.807 |
| 4 | 0.99 | 0.88 | 1.10 | 0.826 |
| 5 | 1.04 | 0.92 | 1.17 | 0.535 |
| SAS #5 Snoring/breathing worse with alcohol | | | | |
| 2 | 1.00 | 0.91 | 1.10 | 0.926 |
| 3 | 1.01 | 0.92 | 1.10 | 0.892 |
| 4 | 1.01 | 0.92 | 1.11 | 0.805 |
| 5 | 1.11 | 1.00 | 1.22 | 0.043 |
| SAS #6 Stop breathing in sleep | | | | |
| 2 | 1.08 | 0.99 | 1.18 | 0.076 |
| 3 | 1.06 | 0.98 | 1.14 | 0.152 |
| 4 | 1.08 | 0.99 | 1.19 | 0.088 |
| 5 | 1.08 | 0.98 | 1.21 | 0.135 |
| SAS #7 Awake unable to breath | | | | |
| 2 | 1.02 | 0.95 | 1.10 | 0.606 |
| 3 | 1.03 | 0.96 | 1.12 | 0.390 |
| 4 | 1.11 | 1.01 | 1.22 | 0.036 |
| 5 | 1.35 | 1.21 | 1.51 | <0.001 |
| SAS #9 High blood pressure |  |  |  |  |
| 3 = don’t know (compared to 1 = no) | 1.16 | 1.09 | 1.24 | <0.001 |
| 5 = yes (compared to 1 = no) | 1.33 | 1.25 | 1.42 | <0.001 |
| SAS #10 Smoking |  |  |  |  |
| 2 = 1 year (compared to 1 = non-smoker) | 1.31 | 1.12 | 1.54 | 0.001 |
| 3 = 2–12 years (compared to 1 = non-smoker) | 1.10 | 1.02 | 1.17 | 0.009 |
| 4 = 13–25 years (compared to 1 = non-smoker) | 0.93 | 0.86 | 1.00 | 0.050 |
| 5 = 26 years or longer (compared to 1 = non-smoker) | 0.99 | 0.91 | 1.07 | 0.762 |
| RR = blood pressure, SAS 1-10 = Items of the Sleep Apnea Score, ESS1-8 = Items of the Epworth Sleepiness Scale. | | | | |
