# Supplementary material for: Risk of Sleepiness-Related Accidents in Switzerland: Results of an Online Sleep Apnea Risk Questionnaire and Awareness Campaigns
Source: Front Med (Lausanne). 2017 Apr 12;4:34. doi: 10.3389/fmed.2017.00034 (PMC5388690; doi:10.3389/fmed.2017.00034)
Supplement: Supplementary file 4 [file Table_4.DOCX]

| **Table S4** Cross-validation results. | | |
| --- | --- | --- |
| Model | Baesian Information Criterion | Median Area under ROC curve (IQR) |
| A. Full model | 54086.51 | 0.73 (0.72, 0.73) |
| B. Full model minus SAS components | 53915.18 | 0.72 (0.72, 0.73) |
| C. Final model (full model minus SAS components and smoking status) | 53883.09 | 0.73 (0.72, 0.73) |
| D. Full model minus ESS components | 57754.13 | 0.63 (0.62, 0.63) |
