# Supplementary material for: Risk of Sleepiness-Related Accidents in Switzerland: Results of an Online Sleep Apnea Risk Questionnaire and Awareness Campaigns
Source: Front Med (Lausanne). 2017 Apr 12;4:34. doi: 10.3389/fmed.2017.00034 (PMC5388690; doi:10.3389/fmed.2017.00034)
Supplement: Supplementary file 5 [file Table_5.DOCX]

| **Table S5** Coefficients for Risk Calculator | |
| --- | --- |
|  | |
| Variable | Coefficient |
|  |  |
| constant | -3.803897 |
| Gender |  |
| female | 0 |
| male | .4922852 |
|  |  |
| BMI^2^ | -.0009926 |
| BMI^3^ | .0000185 |
| Age^3^ | -.0000247 |
| ln(Age)*Age^3^ | 0.00000561 |
|  |  |
| Blood Pressure |  |
| No | 0 |
| Don't know | .1690934 |
| Yes | .3243392 |
|  |  |
| ESS1 (sitting and reading) |  |
| None | 0 |
| Slight chance | -.000832 |
| Moderate chance | .1348893 |
| High chance | .3537316 |
|  |  |
| ESS2 (watching TV) |  |
| Slight chance | -.158775 |
| Moderate chance | -.0122546 |
| High chance | .0218204 |
|  |  |
| ESS3 (public place) |  |
| Slight chance | .268265 |
| Moderate chance | .4664846 |
| High chance | .6504864 |
|  |  |
| ESS4 (passenger) |  |
| Slight chance | .2600178 |
| Moderate chance | .4352554 |
| High chance | .7086098 |
|  |  |
| ESS5 (lying down) |  |
| Slight chance | -.2322735 |
| Moderate chance | -.40239 |
| High chance | -.4309368 |
|  |  |
| ESS6 (sitting and talking) |  |
| Slight chance | .0502038 |
| Moderate chance | .1123082 |
| High chance | .4916046 |
|  |  |
| ESS7 (sitting after lunch) |  |
| Slight chance | -.0056642 |
| Moderate chance | .0721834 |
| High chance | .2008345 |
|  |  |
| ESS8 (in traffic) |  |
| Slight chance | .5779543 |
| Moderate chance | 1.005367 |
| High chance | 1.115103 |
|  |  |
|  |  |
| S = -3.803897 + .4922852(male) - 0.0009926*BMI^2 + 0.0000185*BMI^3 - 0.0000247*Age^3*0.00000561* ln(Age)*Age^^^3 +…. | |
| Risk (100%) = (exp(S)/(1+exp(S)))*100 | |
